# Cancer trends and risk factors in Cyprus

**DOI:** 10.3332/ecancer.2014.389

**Published:** 2014-01-24

**Authors:** Paraskevi A. Farazi

**Affiliations:** Department of Life and Health Sciences, University of Nicosia, 46 Makedonitissas Avenue, P.O. Box 24005, Nicosia 1700, Cyprus; Mediterranean Centre for Cancer Research, UNRF, 46 Makedonitissas Avenue, P.O. Box 24005, Nicosia 1700, Cyprus

**Keywords:** cancer, risk factors, cyprus, cancer incidence

## Abstract

Cyprus, a European Union member state, is a small island in the Mediterranean with a population approaching 900,000 people. Cancer is the second leading cause of death; more therapeutic options for any patient with the disease are available in a central oncology centre in the capital of the island (Nicosia) and fewer therapeutic options (e.g. chemotherapy and hormone therapy only) in a few other public hospitals. Palliative care is offered in several hospices and hospitals, although the field needs improvement. With regards to screening, a national breast cancer screening programme has been in place countrywide since 2007 and is offered free of charge to women between the ages of 50 and 69 years, while colorectal and prostate cancer screening is performed on an individual basis (a pilot programme for colorectal cancer screening was recently initiated). Genetic testing is available for breast and colon cancer. To improve understanding of the causes of cancer in the country, a cancer research centre was established in 2010 (Mediterranean Centre for Cancer Research). Recent epidemiologic work has revealed increasing cancer trends in Cyprus; prostate cancer is the most common in men and breast cancer is the most common in women. Interestingly, thyroid cancer incidence in women has been rising from 1998 to 2008. Cancer of the colon and rectum is also on the rise affecting both sexes. Overall, cancer incidence in Cyprus is lower than other EuroMed countries with similar lifestyle and geography.

## Geography and population

**Figure 1:**
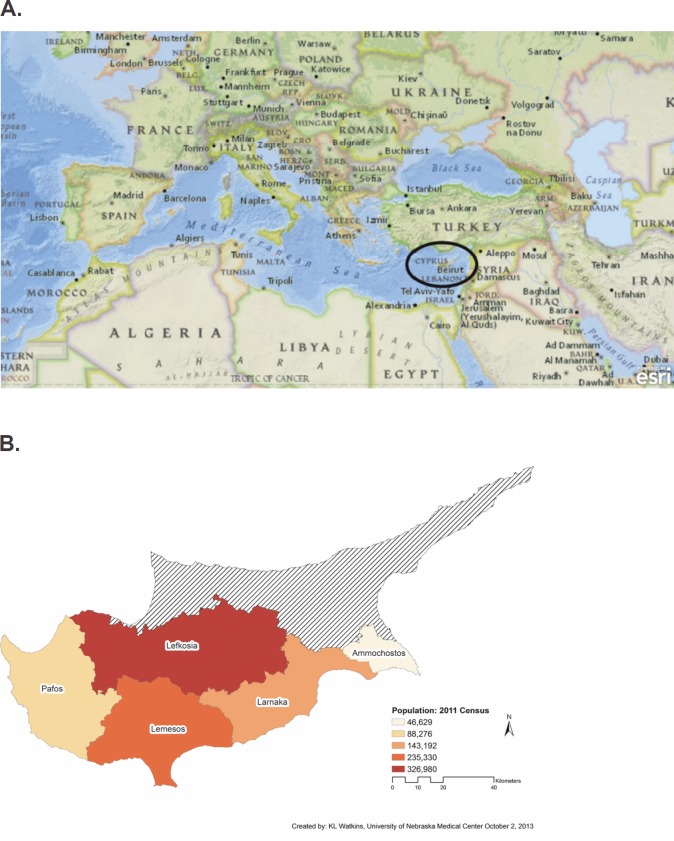
(A) Map showing the geographic location of Cyprus within the Mediterranean Sea. The map was generated using ArcGIS Explorer Online (http://www.esri.com/software/arcgis/explorer-online) Used by permission. Copyright © 2013 Esri and its data providers. All rights reserved. (B) Map showing the five districts of Cyprus. The map was created using ESRI’s ArcMap 10.2 (Redlands, CA), and Turkish occupied boundary was identified using the 2010 Statistical codes of municipalities, communities, and quarters of Cyprus. The population figures are from the 2011 Cyprus Census.

Cyprus is the third largest island in the Mediterranean and is located in the Eastern part of the sea. It has been a member of the European Union (EU) since 2004. It possesses an interesting geographic location as it is at the junction of Europe (360 km southeast of Greece), Africa (300 km north of Egypt), Middle East (90 km west of Syria), and Eastern Asia (60 km south of Turkey) [1] (Figure 1A). According to the 2011 census, the population of the Republic of Cyprus (government controlled area) was 840,407 of which 638,124 were Cypriots (76%; including minorities of Armenians, Maronites, and Latins), 106,561 were people from EU countries (13%), and 90,405 from non-EU countries (11%) [2]. The climate is Mediterranean with dry/hot summers, mild/wet winters, and ample sunshine during the whole year [3]. The island is divided into five districts (Nicosia, Limassol, Larnaca, Famagusta, and Paphos) with Nicosia being the capital of the island (Figure 1B).

## Health

**Figure 2:**
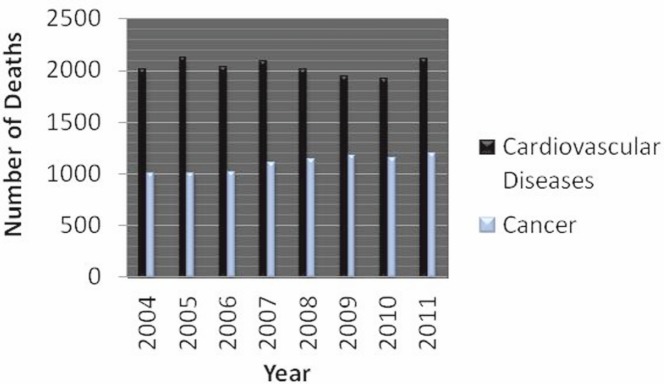
Comparison of the two most common causes of death in Cyprus (2004–2011). Data was obtained from the report for deaths and causes of death of the Cyprus MOH [4].

The life expectancy in Cyprus for 2010–2011 was 79 years for males and 82.9 years for females according to the Ministry of Health’s (MOH) report on deaths and causes of deaths (2004–2011) [4]. Cancer is the second leading cause of death in Cyprus with the first being cardiovascular diseases (Figure 2). The average number of deaths caused by cancer was 1101 in 2004–2011 and by cardiovascular disease 2035. In 2011, cardiovascular diseases accounted for 39.2% of deaths and cancer for 22.1% [4]. However, the increase in the number of deaths in 2011 compared with 2004 was 19% for cancer and only 5.3% for cardiovascular disease, making cancer a significant burden to society. The crude rate of cancer mortality in 2011 was 162.29 for males and 116.02 for females, while the world age-standardised cancer mortality rates were 94.98 for males and 63.33 for females (rates are per 100,000) [4]. According to Eurostat and European Community Health Indicators, cancer death rates in Cyprus are the lowest in the European region [5].

## Major oncology hospitals

Each of the five districts has a government public general hospital that is free for government employees and available to all others at a charge. Nicosia and Limassol hospitals have an oncology department that offers chemotherapy and hormone therapy services and the hospitals of the other three districts only have a visiting oncologist once a week, and offer no chemotherapy services. Patients can also seek advice from private oncologists operating in private hospitals, but with limited therapeutic options. A more complete set of therapies, including radiation therapies, are administered by the single oncology hospital in Cyprus (Bank of Cyprus Oncology Centre; BOCOC, http://www.bococ.org.cy/), which started operating in Nicosia in 1998 and is considered a charity organisation. It was created after a signed agreement in 1992 between the Cyprus government (which donated the land and covers the annual operating expenses of the centre) and the Bank of Cyprus (which provided funds for the construction and equipment of the centre). The BOCOC provides chemotherapy, radiotherapy, hormone therapy, and diagnostic examinations including blood tests and radiological investigations. The two main departments of the BOCOC are radiation oncology/diagnostic radiology and medical oncology. There are specialty oncology units for many different cancer types. It is equipped with three linear accelerators, a superficial treatment unit, an HDR-Brachytherapy system, a simulator, treatment planning systems (3-D), a spiral CT scanner, an ultrasound unit, a mammography unit, a conventional X-ray unit, a SPECT-CT, a picture archiving and communication system (PACS), and computed radiography system. It has facilities, such as two wards for hospitalisation and inpatient treatment (40 beds), a day care unit for chemotherapy (12 beds), a day care unit for supportive care (five beds), outpatient examination/ consulting rooms, an aseptic suite for the reconstitution of cytotoxic drugs, a pharmacy and dispensary, and a laboratory [6].

## Palliative care

Seven institutions currently offer palliative care services for cancer patients in Cyprus [7]. These services are offered by some cancer charity organisations and oncology hospitals. Four non-governmental organisations (NGOs) offer palliative care services: the Cyprus Anti-Cancer Society (which established a hospice in Nicosia in 1976 called Arodaphnousa with the main purpose to offer nursing care to cancer patients dying; in 2000 this hospice officially became a palliative care centre), the Cyprus Association of Cancer Patients and Friends (established in 1986), Friends of the Paphos Hospice (established in Paphos in 2006 within a hospital), and Friends for Life (established in Limassol in 2007). The latter two are newer and provide only palliative hospice care. The first two aforementioned organisations are the oldest and the largest cancer organisations offering palliative care all across the country as well as a more extensive variety of services, such as physiotherapy, psychosocial support, transport service, and so on, all free of charge. They also offer funding for doctors and nurses to obtain special training in palliative care. In addition, palliative care services are offered at two general government hospitals (Nicosia and Limassol) and the Bank of Cyprus Oncology Centre. The drugs that are available for use in palliative care include paracetamol, fentanyl, tramadol, oxycodone, and morphine [7]. Palliative care has experienced tremendous improvements over the last years but it would still benefit by better education of medical professionals regarding pain relief and patients to accept pain relief without fears for addiction and so on.

## Cancer charity organisations

There are several cancer charity organisations in Cyprus whose main goals are to raise cancer awareness and funds to support their services for cancer patients (including palliative care). These organisations include the Cyprus Anti-Cancer Society (http://www.anticancersociety.org.cy/), the Cyprus Association of Cancer Patients and Friends (http://www.pasykaf.org/), Friends of the Paphos Hospice (in Paphos; http://www.paphoshospice.org/), and Friends for Life (in Limassol; http://www.friendsforlife.org.cy/), the Marguerite Cancer Patient Support Team, Europa Donna Cyprus (http://www.europadonna.com.cy/), and Europa Uomo Cyprus (http://www.europauomocyprus.com.cy/).

## Cancer screening

At the moment the only national cancer screening programme implemented is for breast cancer. It was initiated in 2003 in one district (Nicosia) and was expanded countrywide in 2007 (Larnaca and Paphos started the screening programme in 2004, Famagusta in 2006, and Limassol in 2007). It offers free mammography to women of ages 50–69 years old every two years. Records for 2003–2008 show that the response rate of women who received a mammography invitation was 47% for that period [8]. Many women choose to have mammography in the private sector, however, at the moment there are no records on the total number of women who have mammography in the private sector as this information is not collected in any way. An effort is currently underway by the Mediterranean Centre for Cancer Research to obtain such information from all private mammography units since 1998 when the cancer registry has begun its operation. Unfortunately, the task is difficult as not all radiologists are willing to give out that information or their recording system does not allow them to easily retrieve such information. There are 84 mammography units per million women in Cyprus (which makes Cyprus rank as the fifth country among 31 countries with the highest number of mammography units per million women), thus making mammography accessible to women [9]. Interestingly, in a study of ‘cognitive motivations associated with screening mammography in Cyprus’ it was estimated that only 16% of women who were eligible for a screening mammogram in 1997 ever had one [10]. The same study uncovered a quite low score for knowledge of breast cancer screening among women in Cyprus (1.3 in a scale of 0–4) [10]. Another study should be conducted to study, whether the behaviour of women and knowledge about mammography have changed since 1997, especially with the bigger variety of media promoting breast cancer awareness. According to hospital statistics the number of mammograms conducted at public hospitals has tripled in just one decade; whereas, the number of mammograms conducted in 2000 was 8174, the number rose to 24,643 in 2010 suggesting women are becoming more aware of mammography screening [11]. Breast cancer mortality data from 2004 to 2010 indicates no significant reduction in breast cancer mortality since the introduction of the breast cancer screening programme (WASMR was 15.1 in 2004 and 13.1 in 2010), even though it might still be early to note a difference [12].

There is no national cancer screening programme for colorectal cancer and no data for nationwide number of colonoscopies conducted. The only data publicly available exists within the reports of the Nicosia General Hospital (NGH) which show an increase of 62% in the number of colonoscopies conducted in 2011 compared with 2007 at the NGH, indicating an increase in colon cancer screening [13]. A pilot programme for colorectal cancer screening was initiated by the MOH in a rural area of Larnaca (Athienou) last year and invitations have been sent to another community (Aradippou). The plan is to expand the programme to other communities next year (personal communication, Health Monitoring Unit, and Ministry of Health). There is no formal screening for prostate cancer, but PSA testing has been widely used nationwide—a study is underway by the Mediterranean Centre for Cancer Research to investigate the use of PSA testing over the years. No formal screening exists for cervical cancer either. According to the 2003 and 2008 health surveys there was a reduction in the percentage of women who underwent a pap test in 2008 compared with 2003; 80.9% of women surveyed in 2003 reported to have had a pap test whereas only 73% reported the same in 2008 [14, 15]. The cause of the reduction is not clear, however, it warrants further investigation.

## Genetic screening

At the genetic level, various studies have been conducted to explore the frequency of germline mutations associated with breast cancer incidence. Mutation analysis of the BRCA1 gene revealed very low frequency of mutations in the BRCA1 gene in breast cancer patients with a family history of breast cancer [16]. Analysis of a single family with a history of breast cancer incidence in both males and females revealed two BRCA1 mutations (Q356R andS1512I) which may be associated with the breast cancer phenotype in the family [17]. In another study, 29 BRCA2 variants were detected in Cypriot families with family history of breast cancer [18]. A founder mutation in BRCA2 was identified in a different study of Cypriot families with history of breast cancer [19]. Analysis of the BRCA1 and BRCA2 genes in search of mutations in women diagnosed with breast cancer at an early age revealed both BRCA1 and BRCA2 mutations, suggesting that genetic testing for mutations in these genes should be offered to women with early onset breast cancer [20]. Furthermore, single nucleotide polymorphisms (SNPs) in genes involved in the DNA repair pathway (XRCC1 and XRCC2) have been identified in women with breast cancer [21]. Genetic variants of both BRCA2 and the DNA repair gene MRE11A have been associated with the development of breast cancer in Cyprus [22]. Specific haplotypes of the DNA repair genes MRE11A and NBS1 genes were found to be associated with increased breast cancer risk in Cypriot women [23]. Genetic predisposition to colorectal cancer has also been investigated in one study where germline mutations in the APC gene were found in Cypriot patients with familial and sporadic adenomatous polyposis [24].

## Cancer research centres

In 2010, a cancer research centre was established in Cyprus (Mediterranean Centre for Cancer Research, MCCR), which is a non-profit organisation member of the University of Nicosia Research Foundation. The centre aims to perform multidisciplinary research in the field of cancer to better understand the disease and the risk factors associated with it and thus contribute towards cancer prevention [25]. Projects aimed at describing cancer epidemiology and identifying geographic differences in the patterns of cancer development in the island are already underway. Identification of geographic disparities in cancer development is expected to pave the way for further studies to reveal the underlying causes of these disparities, which probably reflect environmental and lifestyle factors. In addition, projects aimed at investigating gene–environment interactions in lung cancer and colon cancer development are being set up. MCCR has been active in establishing collaborations with various institutes and universities in other countries, thus strengthening its research programme. Of course, individual research groups that are dedicated in various aspects of cancer research (e.g. genetics of familial breast cancer, palliative care, and basic research) exist in other institutions such as the Cyprus Institute of Neurology and Genetics, the University of Cyprus, and the Cyprus University of Technology.

## Carcinogenic environmental risk factors

Many environmental factors have been associated with cancer development including smoking, diet, alcohol, various viruses, asbestos, radon, radiation, water and air pollution, and medical conditions whose development is influenced by the environment, such as diabetes and the metabolic syndrome. The way these factors may interact with one another as well as with genetic factors is not fully understood. In Cyprus, even though some of these factors are quite prevalent, their association with cancer has not been investigated. In the subsequent paragraphs the presence of such environmental cancer risk factors in Cyprus is described.

### Tobacco smoking

**Figure 3:**
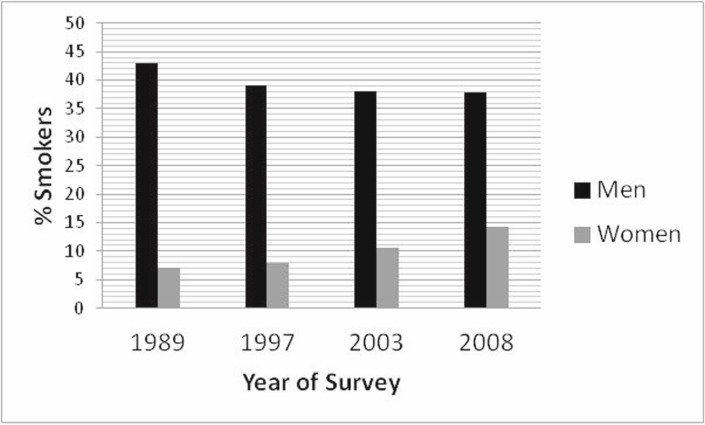
Smoking Prevalence in Cyprus in different years. Surveys were conducted in 1989, 1997, 2003, and 2008.

Tobacco smoking has been associated with the development of many malignancies, such as lung, urinary bladder, oral, pharyngeal, head and neck, and laryngeal cancers [26]. The prevalence of smoking has been investigated in Cyprus to some extent. A survey held in 1989 described a smoking prevalence of 43% among men and 7% for women [27]. In 1997 rates were 39% for men and 8% for women [28]. According to the 2003 and 2008 EU Health Surveys about 38% of males in Cyprus were smokers at the time (for women 10.5% were smokers in 2003 and 14.3% in 2008) (Figure 3). In 2003 Cypriot males ranked ninth among 29 countries in terms of smoking prevalence and women 27th among the 29 countries and in 2008 men ranked fourth out of 16 countries and women eighth out the 16 countries [14, 15]. It is interesting to note that smoking prevalence has remained rather stable in men over time, whereas, it is increasing in women which might translate into more dramatic increases in lung cancer in women in the future. A recent study investigating the cigarette smoking habits of young people has revealed high rates of smoking among young people. Nearly, 37% of high-school boys and 23% of high-school girls are current tobacco users. 52% of the boys that are current smokers smoke every day, whereas 43% of current girl smokers smoke every day [29]. This finding further supports the increasing trend of smoking in females which will change the differences in the incidence of lung cancer among the sexes in the next decades. Considering the high prevalence of smoking, it will be important to assess the impact of passive smoking on cancer development as well. A pilot study has revealed traces of cotinine (metabolised nicotine) in the saliva of 97% of all surveyed children and 94% of children from non-smoking homes (in a survey of 71 households) [30]. In an effort to reduce passive smoking, a smoking ban was introduced in January 2010 in all public places (including bars, restaurants, and so on), even though this is not always respected. However, an evaluation of air quality in several hospitality venues has revealed an improvement in air quality associated with second-hand smoking; the levels of PM_2.5_ associated with second-hand smoking were actually reduced more than 50× (they dropped from 161 µg m^-3^ pre-smoking ban to 3 µg m^-3^ after the smoking ban [31]). In addition, the MOH runs anti-smoking campaigns and smoking cessation clinics in most districts in an effort to reduce smoking [32].

### Diet

Nutrition has been associated with the development of many cancer types [33]. A study among Cypriot children has revealed that 37% of children who were part of a cross-sectional study among 1140 children (average age = 10.7 years) had a poor KIDMED score (poor adherence to the Mediterranean diet which is considered to be a healthy diet) and only 6.7% were high adherers of the Mediterranean diet [34]. According to the 2003 and 2008 European Health Surveys, 33.7% of the population was overweight in 2003 (25<BMI<30) and 12.3% was obese (BMI>30), and these numbers increased slightly in 2008 with 34.1% of the population overweight and 14.8% obese [14, 15]. A case-control study of Cypriot women with breast cancer has shown that a Mediterranean diet rich in vegetables, fish, legumes, and olive oil may favourably influence the risk of breast cancer [35]. It would be interesting, considering the worsening of dietary habits in Cyprus, to see how cancer dynamics will change in the next two decades when the effects of dietary changes and deviations from the Mediterranean diet would exert their effect.

### Alcohol

Alcohol has been associated with cancer and is actually one of the major aetiologies of liver cancer [36]. In Cyprus, the adult per capita consumption of alcohol (years 2003–2005) was 9.3 L, which is <12.2 L which is the average of the WHO European region for that same period and 0.9 L above the average recommended limit of alcohol drinking (8.4 L) according to patient UK [37]. However, it should be noted that an increasing trend in alcohol consumption has been reported for years 2001–2005, which suggests that perhaps the adult per capita consumption might show an increase after 2005 [38]. In a 2007 survey of the European School Survey Project on alcohol and other drugs, it was revealed that alcohol drinking was at moderate levels in Cypriot students compared with other countries. The rate of alcohol use was quite similar (7.9% in Cyprus versus 8.2% in other countries), however, the extent of alcohol use (getting drunk) was much lower in Cypriot students (18%) versus all other countries (39%) [39]. On the other hand, alcoholism has been reported to be on the rise by alcoholic anonymous groups especially among British and other expatriates that have moved to Cyprus; however, no formal study has shown this [40]. The only published information about alcohol drinking in Cyprus comes from the 2003 to 2008 health surveys, which point to an increase in alcohol drinking in more recent years; whereas 59.1% of the surveyed population reported alcohol drinking in 2003, 70.1% reported alcohol drinking in 2008 [14, 15]. However, data on the incidence of alcohol-related disease is missing. Therefore, the association between alcohol and cancer remains to be elucidated in Cyprus.

### Human papillomavirus infection (HPV)

HPV has been associated with the development of cervical cancer and recently a vaccine was developed against two of its strains [41]. There is no published data on HPV infection rates in Cyprus. Since 2010 the Pancyprian Association of Cancer Patients and Friends has been running a campaign for cervical cancer prevention, offering lectures on HPV vaccination as well as a certain number of free PAP Tests and HPV vaccines [42].

### Hepatitis C virus (HCV) infection

HCV infection is one of the major aetiologies of liver cancer in the Western world [36]. A recent study of drug users in Cyprus has revealed 50% prevalence of HCV infection in that population (i.e. 20 of 40 drug users) [43]. Of note, the incidence of hepatitis C cases between 2003 and 2005 was about 0.93/100,000 cases per year [44]. If we estimate that the population in Cyprus around that time was about 700,000 then ~7 cases per year were reported. The aforementioned study revealed ~20 drug users being HCV positive (more than the number of HCV cases in 2003–2005) [43]. If the study was extended to the general population even more cases would be identified, pointing to a sharp increase in HCV infection in the recent years. There is a lag time of a few decades between HCV infection and liver cancer development, thus, the recent increase in HCV infection rates in Cyprus will lead to increased incidence of liver cancer in the coming decades.

### Hepatitis B virus (HBV) infection

HBV infection is associated with the development of liver cancer [36]. In Cyprus, in 1988 the carrier rate of HBsAg was evaluated in different groups of people and was found to be between 0.77% and 1.01% in the group of blood donors and armed forces recruits, 18.27% in family contacts of HBsAg carriers, 6.12% in mentally retarded children, 5.4% in institutionalised adult patients, and 2.94% in hospital personnel [45]. Soon after the publication the MOH made a decision to introduce vaccination for HBV for all neonates, and since the early 1990s HBV vaccination has become standard practice [46].

### Helicobacter pylori (H. pylori) infection

Helicobacter pylori is a bacterium that colonises the stomach and can induce gastric disease including cancer. H. pylori infection has been studied to a limited extent in Cyprus. A study conducted on 103 gastric biopsies revealed the existence of the bacterium in 39.8% of them. The study was confined to only one hospital, and therefore there is a need for a nation-wide study to identify the extent of infection with this bacterium in the general population [47].

### Asbestos

Asbestos is a mineral fibre found in rock and soil and has been associated with lung disease, such as lung cancer, mesothelioma, and asbestosis [48]. In Cyprus, asbestos has been associated with the development of mesothelioma—in some cases in connection with a chrysotile mine in the central mountains of the island and in other cases of asbestos exposure not related to the mine [49]. In 1980, 8% of the population living close to the mine was affected by disease associated with asbestos and in 1990–1995 approximately 30% of deaths in the same area were due to diseases associated with asbestos, such as mesothelioma, asbestosis, and lung cancer [50]. Evaluation of 12 cases of mesothelioma between 1970 and 1980 revealed that five of them were miners of asbestos, three were wives of miners, two were living in nearby villages, and two living further away in Nicosia [51, 52]. This information supports the view that mesothelioma in Cyprus is associated with the mine but also with environmental exposure to asbestos not related to the mine—of note tremolite asbestos was found in the stucco sample taken from houses in nearby villages. A report was prepared for the Ministry of Health on the Health Effects of the Asbestos Mines on the Population of Neighbouring Communities and has shown higher rates of restrictive lung disease among men with occupational exposure to asbestos (41.7% in Kato Amiandos and 28.3% in Kyperounda) compared with women with household exposure (19%) and non-exposed women (4%). Smoking worsened the impact of asbestos on lung disease. There were no statistically significant differences in cancer development among the different groups of asbestos exposure, but the report recommended following up on the issue of cancer development in the next few decades [53].

### Radon

Radon is a product of the breakdown of radioactive uranium, which is found in soil rock and water and has been associated with lung cancer development [54]. Investigations of radon levels in homes and the environment has revealed very low levels of radon in Cyprus. The average outdoor concentration of radon was measured to be 11 ± 10 Bq m^-3^ and average indoor concentration 7 ± 6 Bq m^-3^. The calculated annual dose of airborne radon was found to be roughly 0.19 mSv y^-1^, which is quite low [55]. In another study, measurements of radon using high sensitivity radon portable detectors in buildings and dwellings revealed mean radon concentrations of 19.3 ± 14.7 Bq m^-3^, which is half of the world average of 39 Bq m^-3^ [56]. More recent measurements show passive indoor radon concentrations in Cyprus in the range of 14 ± 3 Bq m^-3^. The same study revealed that the radon concentrations in drinking waters in Cyprus ranged between (0.3 ± 0.3) and (20 ± 2) Bq L^-1^ [57].

### Radiation

Gamma radiation (one type of natural radioactivity) is another environmental factor associated with increased risk for different forms of cancer [58]. High resolution gamma-ray spectrometry was performed indoor and outdoor in different urban locations in Cyprus and revealed that the mean effective dose of gamma radiation to the Cyprus population was 138 µSv y^-1^ which is less than half of the world average of 480 µSv y^-1^. Seal level cosmic rays levels were the same as the world average which is 270 µSv y^-1^ [59]. Exposure to radiation also comes from medical examinations such as X-rays. Interestingly, the number of total X-rays in Cyprus has almost doubled in 10 years; 341,201 X-rays were conducted in all public hospitals in Cyprus in 2000 and this number rose to 588,690 in 2010 [11].

### Water pollution

Water pollution has also been associated with some forms of cancer, most notably digestive cancers in China [60, 61]. A study was conducted back in 2007 to assess the levels of various priority compounds listed in the EU Directive 76/464/EEC on the surface waters of Cyprus. High levels (10 µg/L) of hexachlorobutadiene were found in a few rivers which were actually 100× higher than the quality objective of 0.1 µg/L; hexachlorobutadiene is classified as a potential human carcinogen by the US Environmental Protection Agency [62]. In addition, higher levels compared with quality objective were found for hexachlorobenzene (classified as probable human carcinogen by EPA [63]) and 1,-2,4-trichlorobenzene (listed as not classifiable with respect to its likelihood to cause cancer by EPA [64]) in certain rivers [65, 66].

### Air pollution

Air pollution has been shown to contribute to lung cancer and has actually recently been classified as group 1 carcinogen [67, 68]. A department of air quality exists in Cyprus within the department of labour inspection and the Ministry of Labour and Social Insurance [69]. Air quality is monitored through a network of stations situated in different parts of the island. The department measures the levels of different pollutants emitted, such as NOx, SO2, CO, volatile organic compounds (VOC), benzene, and ozone from traffic, boilers, dry cleaners, hotels, domestic heating, agriculture, petrol stations, and aircrafts [70]. Some of these compounds, e.g. benzene are classified as known human carcinogens by EPA [71]. The levels of benzene, for example, in 2002–2003 exceeded those of the 2010 EU Annual Limit. The levels of benzene emitted due to traffic were higher in Cyprus than many other European cities such as Rouen, Copenhagen, and Munich. The mean values for PM_10_ in Cyprus (2002–2003) were actually higher than many European cities [72]. Since the monitoring of air pollution was established measures have been taken (e.g. strict control of car emissions by forcing all cars to go through the Ministry of Transport Test, which does not allow cars with unacceptable exhaust emissions to be circulating) to reduce air pollution, and hence the levels of these pollutants are expected to decrease in the future. A project called PM3 (part of Life-airquality) is currently underway as a joint collaboration between Cyprus, Greece, and Austria and aims at improving the monitoring capabilities of the air quality department such that sources of pollutant emissions are precisely identified so they can be dealt with accordingly [73]. It would be interesting to see how changing patterns of pollution might correlate with cancer development, especially for cancers of the respiratory system. It is interesting to note that Cyprus also suffers from dust episodes from Sahara (most prominent in the Spring time) when dust from Sahara gets carried with wind currents to Cyprus. It has been demonstrated that Sahara dust carries with it many minerals and microorganisms, which may have an impact on human health [74]. Thus, the Sahara dust might also be a factor contributing to the overall cancer trends, especially to those cancers of the respiratory tract.

### Diabetes and metabolic syndrome

Type 2 diabetes and the metabolic syndrome have been associated with various types of cancer, such as liver, colorectal, bladder, endometrial, pancreatic, and postmenopausal breast cancer [75, 76]. A study conducted between 2003 and 2005 has revealed high levels of diabetes, glucose intolerance, and the metabolic syndrome in Cyprus. The prevalence of diabetes was found to be 10.3%, of impaired glucose tolerance 6.5%, and of the metabolic syndrome 22.2% [77].

### Ultraviolet radiation (UVR)

UVR has been linked to the development of skin cancer [78]. Cyprus has year-round sunshine and therefore UVR levels are on the high end. The population-weighted average daily ambient UVR level for 1997–2003 was 3439 J m^-2^, which was higher than other countries in the Mediterranean region [79].

## Cancer epidemiology

A population cancer registry was established in Cyprus in 1998 when Cyprus joined the Middle Eastern Cancer Consortium (MECC), which was composed of Israel, Palestinian Authority, Egypt, Turkey, and Jordan and was funded by the National Cancer Institute of the United States. According to the MECC monograph which described cancer incidence in four MECC member countries (1998–2001 for Cyprus) the age standardised incidence rates of all cancers in Cyprus (164.2) were comparable with those of Israel (Arabs) and Egypt, were higher than Jordan and significantly lower than Israel (Jews) and US SEER [80].

**Figure 4:**
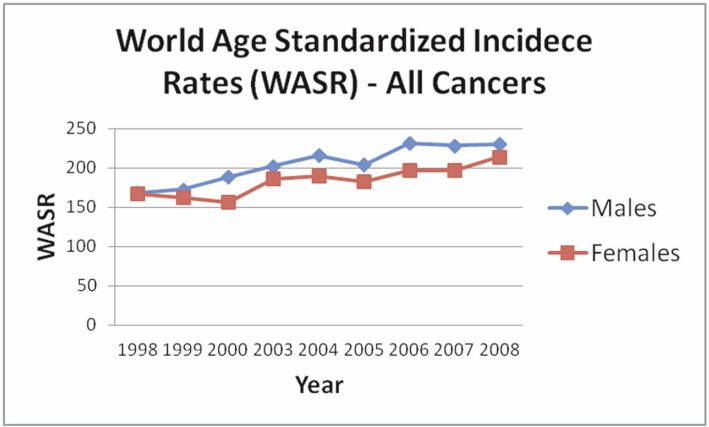
Increasing incidence of all cancers in males and females from 1998 to 2008. Data was obtained from the cancer reports of the Cyprus MOH. Note that data for 2001 and 2002 was not available in these reports.

**Figure 5:**
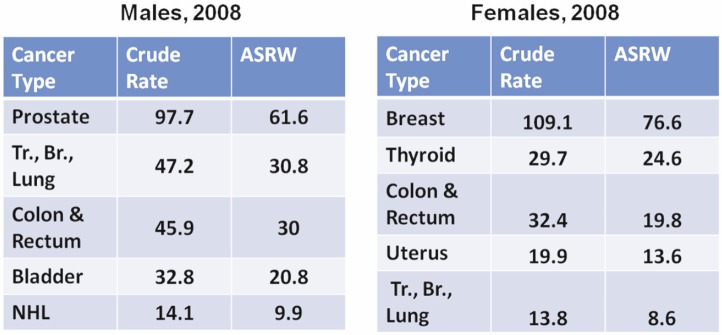
Crude and world age-standardised rates of the five most common cancer types in males and females in Cyprus (2008).

Finalised cancer registry data exists until 2008 and shows that overall cancer incidence has been increasing since 1998 (Figure 4). The latest analyses of cancer incidence for 2008 revealed that prostate cancer is the most common cancer type in men, followed by trachea/ bronchus/lung cancer, colorectal cancer, bladder cancer, and Non-Hodgkin’s lymphoma (NHL) (Figure 5) [81]. Colorectal cancer in males is increasing to the point it looks it might actually surpass trachea/bronchus/lung cancer and become the second most common cancer type in men. Interestingly, the rates of lung cancer in men (ASRW = 30.8 for 2008) are relatively low compared with other EuroMed countries (e.g. Spain Granada; ASRW = 54.7 for 1998–2002 according to Cancer Incidence in Five Continents results [82]), which is surprising considering a 43% prevalence of smoking in Cypriot men in 1989 [27]; unfortunately older data on smoking is not available, and therefore there exists the possibility that a potentially lower prevalence of smoking in the 1960s and 1970s might account for the overall lower lung cancer incidence in Cyprus. There is a lag time of a few decades between smoking and the development of cancer, therefore, data on lung cancer in the coming decades and better evaluation of smoking trends a few decades ago will resolve this question.

In Cypriot women, breast cancer was the most common cancer type in 2008, followed by thyroid, colon and rectum, uterus, and trachea/bronchus/lung cancer (Figure 5). The rates for thyroid cancer have doubled in just one decade in women, which warrants further investigation into the causes of the increase (either higher detection due to frequent performance of fine needle aspiration or true increase due to some sort of radiation exposure). It has been demonstrated that access to better care is influencing the rates of thyroid cancer by overdiagnosis of this disease [83]. Since breast cancer is the most frequent cancer type in women, risk factors for the disease in the Cypriot population have been studied and it was shown that family history of breast cancer is the strongest predictor of breast cancer risk and late menarche and breastfeeding exhibit a protective effect in Cypriot women [84].

Interestingly, tobacco-related cancers show lower incidence in Cyprus compared to other neighbouring countries, the UK and USA (Table 1). Tobacco-related malignancies include lung (including trachea and bronchus), urinary organs (including renal pelvis, ureter, bladder, and other urinary organs), kidney (renal cell), pharynx (including tongue, mouth, tonsil, hypopharynx, pharynx unspecified and other oropharynx, nasopharynx, nose, sinuses and so on), and larynx. Whether this lower rate reflects lower smoking prevalence, or lower exposure to other carcinogenic environmental factors, and/or genetic factors is not known.

Despite the high UVR levels in Cyprus, skin cancer rates are relatively low; in 2008 the WASR of melanoma was 5.8 for men and 5.7 for women. Melanoma of the skin was the eighth most common cancer in both men and women in 2008 [78]. Whether the rate reflects better protection from UVR through human factors (use of sunscreen and protective clothing during exposure to the sun) or genetic factors (relating to skin colour etc) is not known. Considering the geographic location of Cyprus and conquests it experienced by many different populations, such as Phoenicians, Egyptians, Romans, Venetians, Ottomans, British, and so on [85], the genetic make-up of the Cypriot population is most likely complex and reflected in the enormous skin colour variation of the people (going from very light complexions to very dark complexions). Even though the rate of skin cancer is relatively low, it has been rising over the years suggesting UVR is having a bigger impact on the population.

Finally, it is interesting that the overall cancer rates in Cyprus are lower (ASRW = 249.9 for men and 225.7 for women in 2008) compared with other EuroMed countries (e.g. Spain Granada; ASRW = 400.4 for men and 252.4 for women for 1998 – 2002 according to Cancer Incidence in Five Continents results [84]) which share a similar geographic location and lifestyle and the reasons for this are not known. To get a better picture of the cancer trends over the years in Cyprus, an epidemiologic study was recently conducted by MCCR in collaboration with the University of Michigan School of Public Health, University of Nebraska Medical Centre, and the Cyprus Ministry of Health using cancer registry data from 1998 to 2008, the results of which will soon become available.

## Conclusions

Cyprus is a small country with interesting trends in cancer incidence. Its small size makes it a unique setting for understanding genetic and environmental contributions to cancer development. It provides many opportunities for carefully designed case-control and prospective cohort studies since distances are quite small and the population can be easily followed up. It will be interesting to see how the cancer trends change over time in the future, especially since many environmental factors are changing such as higher rates of hepatitis C infection due to drug abuse, higher incidence of the metabolic syndrome, lower adherence to the Mediterranean diet, and increased smoking trends (especially in women) in the recent years.

## Conflicts of interest

The author has no conflicts of interest to declare.

## Figures and Tables

**Table 1.** Age standardised average annual incidence rates per 100,000 for selected tobacco-related malignancies, by country based on Globocan 2008 estimates and CI5 (http://ci5.iarc.fr/CI5i-ix/ci5i-ix.htm).

		MOH (WASR)	Globocan 2008 (ASR)						
		Cyprus	Cyprus	Greece	UK	Spain	Italy	Turkey	Israel
Oral cavity	M	4.7	3.5	4.1	9.1	17	9.5	5.6	5.9
	F	1.5	2.3	1.5	4	3.7	3.3	2.4	2.7
Larynx	M	5.8	2.3	4.2	3.5	9.5	7.3	8	5
	F	0.2	0.5	0.3	0.6	0.4	0.6	0.5	0.8
Lung	M	30.8	22	52.2	38.2	53.3	45.4	49.1	33.1
	F	8.6	4.9	9.5	25.9	7.7	11.4	5.2	16.1
Kidney	M	5	4.6	5.6	10.4	8.4	10.3	3.5	13.3
	F	3	2.3	2.3	5.3	3.5	4.8	2	5.6
Bladder	M	20.8	16	15.2	11.7	27.7	20.1	16.1	29.7
	F	3.9	3	2.7	3.3	3.2	3.5	2.3	5.2

**Table:** 

		MOH	CI5				
		Cyprus	Turkey Antalya	Italy Naples	Spain, Granada	Israel	USA SEER
Lung	M	24.8	37.5	66.2	43	32.2	52.8
	F	5	4.8	10.2	3.3	12.1	34.5
Larynx	M	3.2	7.5	11.7	11.2	4.9	4.9
	F	0.3	0.6	1.1	0.2	0.7	1.1
Pharynx^a^	M	3.1	3.5	6.1	11.1	4	9.6
	F	1.7	1.7	1.8	2.3	2	3.8
Kidney	M	5.1	3.3	7.8	6.7	12.3	11.9
	F	2.8	1.9	5.1	3.1	6.1	6
Urinary organs^b^	M	20.7	15.8	47.2	30.2	27.7	20.9
	F	3.5	2.5	6.4	3	5.1	5.4

a Pharynx—here, including only tongue, mouth, pharynx, nose, and sinuses.

b Urinary organs—here, including only urinary bladder.
